# Roles and relationships between health professionals involved in insulin initiation for people with type 2 diabetes in the general practice setting: a qualitative study drawing on relational coordination theory

**DOI:** 10.1186/1471-2296-15-20

**Published:** 2014-01-31

**Authors:** Jo-Anne Manski-Nankervis, John Furler, Irene Blackberry, Doris Young, David O’Neal, Elizabeth Patterson

**Affiliations:** 1 General Practice and Primary Health Care Academic Centre, University of Melbourne, Carlton, Victoria; 2 Department of Medicine, St Vincent’s Hospital, Fitzroy, Victoria; 3 Department of Nursing, Melbourne School of Health Sciences, University of Melbourne, Carlton, Victoria

**Keywords:** Relational coordination, General practice, Insulin initiation, Type 2 diabetes, Roles

## Abstract

**Background:**

The majority of care for people with type 2 diabetes occurs in general practice, however when insulin initiation is required it often does not occur in this setting or in a timely manner and this may have implications for the development of complications. Increased insulin initiation in general practice is an important goal given the increasing prevalence of type 2 diabetes and a relative shortage of specialists. Coordination between primary and secondary care, and between medical and nursing personnel, may be important in achieving this. Relational coordination theory identifies key concepts that underpin effective interprofessional work: *communication* which is problem solving, timely, accurate and frequent and *relationships* between professional roles which are characterized by shared goals, shared knowledge and mutual respect. This study explores roles and relationships between health professionals involved in insulin initiation in order to gain an understanding of factors which may impact on this task being carried out in the general practice setting.

**Method:**

21 general practitioners, practice nurses, diabetes nurse educators and physicians were purposively sampled to participate in a semi-structured interview. Transcripts of the interviews were analysed using framework analysis.

**Results:**

There were four closely interlinked themes identified which impacted on how health professionals worked together to initiate people with type 2 diabetes on insulin: 1. Ambiguous roles; 2. Uncertain competency and capacity; 3. Varying relationships and communication; and 4. Developing trust and respect.

**Conclusions:**

This study has shown that insulin initiation is generally recognised as acceptable in general practice. The role of the DNE and practice nurse in this space and improved communication and relationships between health professionals across organisations and levels of care are factors which need to be addressed to support this clinical work. Relational coordination provides a useful framework for exploring these issues.

## Background

“Shared doctoring requires that everyone concerned should take each other’s contributions seriously and at the same time attune to what bodies, machines, foodstuff and other relevant entities are doing. Those who share doctoring must respect each other’s experiences, while engaging in inventive, careful experiments. They must attune all variables to each other, while attending to everyone’s strengths and limitations. They must change whatever it takes, including themselves” Annemarie Mol [1].

Mol highlights the critical and intimate link between health professionals, their roles and the individuals occupying these roles and its importance for achieving quality, personalised outcomes for patients. Studying the work involved in insulin initiation for people with type 2 diabetes (T2D) provides a lens for exploring the factors that may influence how effectively generalist and specialist health professionals across both primary and secondary care work together to improve health outcomes. Improving integration between health professionals and services is one of the five building blocks for reform under Australia’s National Primary Health Care Strategy [2,3]. Delivery of best practice care in the primary care setting requires collaborative and coordinated practice and this is dependent on effective interprofessional relationships [4]. While such relationships are likely to be influenced, in part, by gender roles and historical factors between doctors and nurses and generalists and specialists [5-7], advancing collaborative practice also requires attention to the more proximal and immediate factors that shape how professionals communicate and coordinate their work.

The aim of the research reported in this paper was to use an organisational theoretical framework to explore the roles and relationships between health professionals involved in the initiation of insulin for people with type 2 diabetes in the general practice setting to provide a better understanding of how multidisciplinary care works in practice.

### Type 2 diabetes and insulin initiation in general practice

Type 2 diabetes (T2D) is a chronic medical condition characterised by increased blood glucose levels which result from reduced or less effective insulin and is associated with significant complications including renal failure, blindness, heart attack, stroke, nerve damage and peripheral vascular disease [8]. One component of T2D treatment is to optimise blood glucose levels. At 10 years post-diagnosis approximately 50% of people with T2D will require exogenous insulin to maintain optimal glycaemia [9]. In Australia, the majority of care for people with T2D occurs in general practice [10]. However, when insulin initiation is required it is generally not occurring in a timely manner [11-13] and it has been shown that primary care physicians are more likely to delay initiating insulin compared to specialist colleagues [14]. Despite evidence that it is safe to initiate insulin in general practice [15-17] and it being a core general practice activity in countries such as the United Kingdom [18] and the Netherlands [19], in Australia the majority of patients are referred to specialist care for insulin initiation [20]. However, the relative scarcity of these health professionals [21-23] can result in delays to starting this treatment. Increasing the capacity for general practice to initiate initiation will be important to achieve timely insulin initiation, especially given the increasing prevalence of T2D. Support and coordination of care, together with effective communication between primary and secondary care are likely to be important factors in facilitating this.

### Health professionals involved in insulin initiation

With increasing knowledge and technology it is not possible for any one health professional to effectively and efficiently provide the spectrum of care required by patients with chronic conditions, such as T2D and so multidisciplinary coordinated care is recommended [24-26]. Health professionals most commonly involved in insulin initiation in Australia include general practitioners (GPs), diabetes nurse educators (DNEs) and specialist physicians. In other countries practice nurses (PNs) (nurses who work with and under the supervision of GPs) play a significant clinical role in insulin initiation and the potential for this to occur in the Australian setting is currently the focus of a cluster randomised controlled trial in Victoria, Australia [27]. In Australia PNs do not require any formal postgraduate qualifications but have been required to meet continuing professional development standards since July 2010 [28]. Credentialed DNEs are also required to meet these standards, however they have also completed a Graduate Certificate course (1 year part time), 1800 hours experience in facilitating diabetes self management and providing education and have completed a mentoring program [29]. They may work in primary care, secondary care settings or both, but are considered specialists in their area.

As Mol [1] suggests, if PNs are to take on an expanded role in insulin initiation the health professionals currently involved in this task may need to reflect on how they can support this, even if this impacts on their current practices. Established roles and relationships between these health professionals may influence their willingness to commit to this support.

### Relational coordination

Relational coordination theory provides a possible framework for exploring the roles and relationships between health professionals involved in the initiation of insulin.

Relational coordination is defined as “a mutually reinforcing process of interaction between communication and relationships carried out for the purpose of task integration” [30]. This theory, first developed by Gittell to explain the impact of role relationships on coordination and organisational outcomes in the aircraft industry, has now been applied in multiple health care settings, including primary care, and a survey tool developed to facilitate its measurement [30-33]. Relational coordination theory identifies key concepts that underpin effective interprofessional work: *communication* which is problem solving, timely, accurate and frequent and *relationships* between professional roles which are characterized by shared goals, shared knowledge and mutual respect [30].

## Method

Twenty one GPs, PNs, DNEs and specialist physicians were purposively sampled from 179 respondents to a survey in which relational coordination between health professionals involved in insulin initiation was measured [34]. In this survey 58% of DNEs, 37% of GPs, 34% of PNs and 37% of specialist physicians consented to being contacted regarding participating in an interview. Maximum variation sampling was utilised to capture a wide range of perspectives and experiences. Two to three health professionals of each type above and below the median relational coordination score were purposively selected to participate in an interview to explore their current professional roles, working relationships with other health professionals and practice or organisational factors impacting initiation of insulin in the general practice setting. Where possible health professionals were also selected on the basis of gender, the type of clinic they worked in (e.g. hospital or general practice) and at least one health professional per group was from a regional or rural setting. This sample size is in keeping with those typically seen with purposive sampling, which usually consists of 30 cases or less [35]. The aim of these interviews was not to obtain saturation, generalisability or to link responses to individual scores, but rather to explore possible factors underlying the relational coordination domains in this purposively selected group.

Interviews were conducted either face to face or via telephone if this was requested by the participant. The interviews were semi-structured according to a pre-written interview schedule which was flexible, updated as the study progressed and allowed exploration of the factors underlying the interviewee’s responses. All interviews were conducted by the first author (a female GP). Interviews took between 30 and 45 minutes, were digitally recorded and then transcribed by a professional transcription service. The transcript was reviewed and then uploaded into NVivo 9 (QSR International) for framework analysis, a matrix based method for ordering and synthesising data [36,37]. Framework analysis was developed by Ritchie and Spencer at the National Centre for Social Research, United Kingdom, in the 1980s [36,37]. Analysis consists of six stages: familiarisation, identification of descriptive categories, indexing, charting, investigation and interpretation and report findings [37].

Each transcript was entered as a case node and then characterised according to gender, health professional type (GP, DNE, PN, specialist physician), the health care setting in which the participant worked (general practice, private, hospital, community health centre), duration of practice, geographical (RA) classification and relational coordination survey scores.

The key themes were initially based on relational coordination, collaboration and factors impacting on these as determined from a literature review and new themes were identified as the interviews were conducted and analysed. These were used to build the index for the study which included barriers and facilitators to insulin initiation, education and training, intra and interprofessional relationships, health care setting, professional roles, trust and relational coordination domains between health professional dyads. Key points identified within the transcripts were summarised (charted) into the framework with mapping to the original text in the transcripts (indexing). Two interviews were reviewed with the second author, a GP qualitative researcher, and results compared. The purpose of this was not to reach concordance but rather to introduce different viewpoints regarding coding and interpretation of the data and to refine the coding and analysis [38]. Comparisons were made within and between different health professional groups and common and contrasting themes were developed.

This study received ethical approval from the Human Research Ethics Committee (HREC) at The University of Melbourne (HREC ID: 1238199). All participants provided written consent prior to participating in the interview.

## Results

Twenty one health professionals were interviewed between July 2012 and February 2013 (see Table 1 for summary of characteristics). Four main themes relating to roles and relational coordination between the health professionals involved in the task of insulin initiation were identified. Quotes relating to each of these themes are detailed in Table 2.

**Table 1:** Characteristics of interview participants

	**Code**	**Gender**	**Years in practice**	**Type practice**	**Practice location (Primary RA level)**	**Total relational coordination score (relation to median for health professional group)**	**Interview type**
**Physician**	PHY503*	Female	7	Hospital	1	3.25 (<med)	Face to face
	**PHY509**	Male	30	Hospital and private office	1	4.00 (> = med)	Face to face
	**PHY511**	Female	11	Hospital and private office	1	3.80 (> = med)	Face to face
	**PHY512**	Female	1	Hospital and community health centre	1	4.29 (> = med)	Face to face
	**PHY518**	Male	3	Hospital	1	3.54 (> = med)	Face to face
	**PHY527**	Male	30	Hospital and private office	2	3.57 (> = med)	Face to face
**DNE**	**DNE102**	Female	10	Community health centre	2	4.29 (> = med)	Face to face
	**DNE108**	Female	8	General practice	1	3.00 (<med)	Telephone
	**DNE112**	Female	15	Hospital	2	3.25 (<med)	Telephone
	**DNE134**	Female	25	General Practice	2	4.64 (> = med)	Telephone
	**DNE148**	Female	3	Community health centre	1	3.54 (<med)	Face to face
**GP**	**GP714**	Male	7	General practice	1	2.86 (<med)	Face to face
	**GP724**	Female	25	General practice	2	3.29 (<med)	Face to face
	GP730*	Female	2	Community health centre	1	3.46 (<med)	Face to face
	**GP744**	Male	20	Community health centre	1	3.89 (<med)	Face to face
	**GP745**	Male	25	General practice	1	3.75 (> = med)	Face to face
**Practice nurse**	**PN412**	Female	3	General practice	1	3.00 (<med)	Face to face
	**PN415**	Female	5	General practice	1	4.04 (> = med)	Face to face
	**PN417**	Female	10	Community health centre	1	3.21 (<med)	Face to face
	**PN418**	Female	10	General practice	2	3.06 (<med)	Face to face
	**PN423**	Female	8	General practice	1	4.86 (> = med)	Face to face

*Registrar.

**Table 2:** Quotes relating to identified themes

**Theme**	**Quotes**
***Ambiguous roles***	*DNE108: How long before GPs say well okay, we’re using the practice nurses to start glargine, why don’t they do this and why don’t they do that? Is the aim of this to put CDEs out of a job…That’s where I have my real concern is that if GPs start using practice nurses to start glargine that this is the very first step. I understand clinics that can’t get CDEs, that they can’t get these people to put on to insulin and things like that. Look, I understand that there are clinics that have this and that’s – that’s our role in the CDE is to get CDEs into these clinics rather than using practice nurses who have very, very limited education and understanding in that area.*
	*PN415: The general gist is what you get for prac nurses is when are you going back to real nursing, is everything that you get. Or you must be really bored, you must not do anything. People’s perception of practice nurses is you sitting on your backside drinking coffee and doing blood pressures unfortunately. Usually - that’s what I said, in practice nursing you can be as proactive or not as proactive as you like. So it really depends on the nurse, the team you’ve got and the things within your facility and your reach that you can reach out to.*
	*PHY527: Practice nurses would interrelate with your diabetes nurse eds and all that and would probably reduce their load as well. Because it’s another set of eyes and ears if you educate them appropriately.*
***Uncertain competency and capacity***	*DNE148: So with practice - I think some practice nurses do believe they’re - but it’s a personality type - they’re above and beyond and they can manage everything. Why did we (DNEs) go and spend $10,000 to get a qualification that reaps us no rewards at the end of the day? Apart from the fact we don’t need to wear a uniform at some places. But that’s their personality, that they think they can do stuff. Within their nurse role they should be able to titrate insulin. It’s a drug like any other drug. They should know how to do it. But do they have the competence to do it, that’s debatable, and do they understand the mechanisms behind it? Sometimes that will be a challenge but as long as they know they can ask for help then there’s no issue.*
	*PHY509: Well, I mean - I think increasingly [practice nurses] are doing sorts of work like - they are preparing the patients in between consultations - I don’t know, maybe weighing them, taking their blood pressure, maybe assisting people with teaching them other various things. I mean, I’m sure that practice nurses do a different series of jobs. But not - see, what’s being said now is that the practice nurse can be doing all sorts of diabetes education activities. I don’t know to what degree they’re doing that - so, yeah - I mean, I don’t know whether there’s a job description that far, that covers all practices, I doubt it.*
***Varying relationships and communication***	*PHY503: What I found the most helpful is when we’ve had a couple of really tricky patients, we just do all our consults together, me and the educator, just see them together so you’re all on the same page and it’s more of a team thing and try and have it as a conversation between three people rather than being too didactic… There was quite a few times when I saw people with the educator, that crowd, they’re mainly older decrepit people basically. That was really helpful and I find that you’re doing it together, even the first consult you do that together because you can be asking a question, both of you ask questions and if you forget something, you get the full picture and you both know what’s going on.*
	*PHY509: But, I mean, I would hope that we – we don’t - we honestly don’t deliberately try and hold on to people but it’s just in this context of where you never hear anything back and you don’t - and people come back and you’ve suggested various things to get done and that hasn’t been done. You don’t know whether it’s because the patient didn’t do it or because the doctor didn’t do it or because of a whole lot of reasons. So, you tend - in that sort of context, your tendency is to say, okay well - no, we’ll do this - we’ll do this here now and…See you again to see what’s wrong.*
	*GP744: The difficult stories are the patients who go to the [public] Hospital or something like that in the public. They’ve got type 2 diabetes; we get the most dreadfully pointless, vanilla reports back. We don’t - there’s this kind of out of touch process.*
	*DNE148: So we sort of know people in common. But you have to go to sort of the company dinners to meet the other endocrinologists and the other GPs, just so they know who you are and what you’re doing. Then you’ll get a better response….[Do the endocrinologists interact with you differently compared to the GPs?] Absolutely…I think they - or the ones I’ve dealt with anyway - see us as part of the team. I guess I’m fortunate I work with good people but you’re there, you’re the one that gets the difficult patient. You’re the one that has to speak to them about their diet and exercise every month for the next six months until you get through to them. So they can see that there’s our role and there’s their role and most of them are quite flexible about what’s what and who’s who. So you work together as a team…*
***Developing trust and respect***	*DNE148: [Do you think the GPs trust you?] Some do. If I didn’t - if I hadn’t have worked for so long with this one cranky-pants GP, I would think it would personal. But it’s not, it’s their mindset. As much as you know some people can’t drive Fords or Holdens, it’s no, diabetes nurse educators are not worth anything. So it’s not personal and if you can explain it as a professional, sometimes they don’t know - I guess diabetes educators can be nurses, they can be podiatrists, dietitians, they can be dentists. So they don’t know what angle you’re coming from but once you identify you’re a nurse and this is what happens and this is how it goes, then there’s a bit more respect.*
	*DNE102: So I suppose I don’t have as much of that battle that a lot of nurses in hospitals have with the doctors. It’s probably because I’m older as well maybe, I don’t know, and also it’s your relationship with them - in terms of how you respond. It’s not like I want you to, it’s would you consider? There are ways of talking and communicating in a way that they feel like they’re making the decision. [So you still feel that you need to do it that way?] Only with some. The others I will just - they’ll just ring or they’ll send them in and say just do whatever [the DNE] asks sort of thing. There’s no point anyway, she’s going to change it they go.*	
	*GP730: I think - certainly in the way that [DNE] practiced, so she would review patients and have it sort of scheduled in, she was almost acting like an endocrinologist in terms of her suggestions and her knowledge about therapies and what was appropriate. So she - to some extent, her experience vastly outweighed pretty much every GP at the practice and her knowledge base was such that we all felt comfortable with her suggesting treatment options and guidelines which we would then put in place.*	
	*PN415: Because I understand when you’ve taken the initiative and you’re doing a course and - I’ve looked at the diabetes course and the credentialing and I’ve had friends and I’ve been with them while they’ve gone through it. It is a gruelling process. It is a lot of - a lot to take on in a year and then being credentialed and then how you - what you have to do to stay credentialed. So yes they are specialised in that.*	

### Ambiguous roles

In general there was good agreement among all health professionals as to the role of specialists in the care of patients with T2D who required initiation of insulin. For example, all generally agreed that the specialist physician’s role should be to review and manage people whose diabetes was complex because of co-morbidities, those who had secondary causes of their diabetes, or who did not attain optimal blood glucose levels in the general practice setting. DNEs expressed that their skills in diabetes education, insulin initiation and titration were superior to other health professional groups, and this was reinforced by all groups, especially specialist physicians. This shaped how they viewed their role, which was central to providing high quality, safe, appropriate care to patients.

There was less consistency when it came to the roles of generalists (GPs and PNs), and these views particularly differed between those in specialist roles and those in primary care. For example, the GPs interviewed believed that management of T2D, including insulin initiation, was core work for general practice. One PN was currently involved in diabetes education, insulin initiation and titration and others were in the process of working towards developing these skills to further develop their role in T2D management. GPs saw the PN role as complementary to their own and that with adequate training they should be able to manage uncomplicated type 2 diabetes together, including initiating and managing insulin if there were clearly defined guidelines and protocols.

There were some opposing views amongst those in specialist care whether PNs could, or should, have a role in insulin initiation. This was particularly the case for DNEs, some of whom viewed this as a potential threat to their role. Whilst some saw insulin initiation as the domain of GPs and DNEs, others thought GPs could do this in partnership with PNs if there was DNE support. In contrast, specialist physicians felt that insulin initiation was a role that GPs could undertake but like them, should have the back up and support of a DNE to do this.

The lack of support for a role for the PN by some may reflect a lack of understanding of the PN role. PNs generally felt that DNEs didn’t fully understand their role with one reflecting that ‘People’s perception of practice nurses is you sitting on your backside drinking coffee and doing blood pressures’, and that PNs are not a ‘real nurse’. They felt that this may in part be because of diversity in PN roles and a lack of career structure. This variety is dependent on the proactivity of nurse, the team and setting in which they work.

### Uncertain competency and capacity

Concerns about competency and capacity were closely linked to the discussion of roles.

The expansion of the PN role was seen to provide opportunities for insulin initiation for people with uncomplicated T2D in the patient’s home practice and freeing up DNEs to be able to see more complex patients if the PN had adequate training and mentoring and support from a DNE for less complex patients. However, others had safety concerns secondary to a perceived lack of knowledge on the part of the supervising GPs, inadequate PN training, inadequate time given their multiple other roles within the practice and likely low frequency with which initiations would occur.

Whilst specialist physicians felt that PNs could play a role in insulin management they shared some of these concerns. This is likely to reflect the uncertainty that some specialist physicians had as to the degree to which PNs undertake any diabetes-related activities and an inability to identify which PNs are involved in diabetes care.

### Varying relationships and communication

The strongest relationships and communication occurred within levels of care. For instance, specialists (specialist physicians and DNE) reported good working relationships which were characterised by shared knowledge and acceptance of each other’s roles. Mutual respect and trust which developed early in careers was facilitated by ongoing contact and communication. Overall, DNEs and specialist physicians had an understanding of who does what, how and when to communicate. However, for some specialist physicians this contact and communication appeared to be reduced across levels of care, with resultant lack of relationships with community based DNEs as compared to those working in hospitals.

Similarly, GPs and PNs relationships developed over time and were facilitated by knowledge of each other’s roles, boundaries and close observation of each other’s work. Frequent, timely and accurate communication was facilitated by collocation and shared medical records as well as opportunities for problem solving communication in clinical meetings and case conferences. For GPs these relationships developed in part from a need for self preservation and to prevent ‘burn out’, and as a result these GPs were supportive of the PN role and their ongoing skill development including insulin initiation. The GPs saw this as essential because it was otherwise impossible to carry out all the administrative tasks associated with general practice and provide optimal patient care as well. However, some acknowledged that it took time to adjust, develop trust and feel comfortable with PNs taking on an extended role. In this way the relationship was not as developed as that between specialist physicians and DNEs.

DNEs in community health centres and private practice potentially act as a link or spanner^a^[1] between the two levels of care, particularly where there was collocation. GPs expressed that lack of access to DNEs could result in barriers to collaboration and problem solving communication. However, DNEs generally provided timely communication regardless of where they were located. Whilst relationships between DNE and PN were generally seen as important the existence of these was variable and once again depended largely on face to face meetings and collocation which facilitated all domains of communication.

For GPs relationships with specialist physicians varied depending on whether the specialist physician was working privately or in the public hospital outpatient department. There was limited opportunity for these professionals to meet face to face and hence their view of each other was based on responsiveness to referrals and communication. GPs said that they preferred to refer patients to private specialist physicians about whom they had received positive patient feedback and had an understanding of how they worked with patients. In contrast, specialist physicians working in hospital outpatient clinics were described as ‘faceless’ doctors who were not chosen or known and with whom there were issues with timely clinical intervention, timely and problem solving communication. Despite these concerns, GPs had no choice but to access this service because of patient’s inability to afford private care.

Because of high workload, reduced communication and a lack of knowledge of GP skills and interest, specialist physicians expressed that the only way to assess GP knowledge and skill was through their referral letters and they commented that there was variation in the quality, accuracy and completeness of these in regards to history, medications, pathology results, what the GP would like them to do and the other health professionals involved in care. DNEs also expressed similar problems with accessing information from GPs. Whilst GPs commented about a lack of information/communication from hospitals one specialist physician expressed a sense of frustration that they never heard back from GPs unless they were complaining about the service. This lack of problem solving communication could result in a lack of trust and a feeling that the specialist physician needed to take over care.

### Developing trust and respect

Similar to PNs, DNEs felt that most GPs respected and trusted them in their role as a result of them seeing and experiencing their knowledge and competence and having experienced successful management with DNE involvement. However, some perceived a need for careful communication and negotiation with GPs in order for GPs to accept their management advice or recommendations and continue to work with them. This was described by one DNE as ‘manipulation’. It was felt that some GPs had a professional mindset not to trust nurses and those doctors were unable to accept that some nurses could have more knowledge than them. The DNEs interviewed had variable respect for GPs. One DNE viewed GPs as knowing a little about a lot with little knowledge about diabetes management and insulin, and slow to take up management advice. Those that were perceived as proactive were more respected. However, the concern that GPs may use PNs to substitute for the DNE resulted in a feeling that their knowledge and training was not respected or acknowledged. Despite this the GPs interviewed in this study said that they appreciated DNEs’ knowledge and expertise and understood their role and because of this relied on them to assist in management. One GP equated the DNE as equivalent to an endocrinologist in terms of their knowledge and expertise.

Despite some DNEs feeling the PNs didn’t respect their training or expertise, PNs described respect for the DNE role and their training and credentialing which was seen as a gruelling process - and those that had access to a DNE felt that they were lucky to do so.

## Discussion

The increasing burden of T2D in Australia and a relative lack of specialists means that increased care for people with T2D requiring insulin needs to occur in general practice. In this study there was support for this to occur, although some participants were guarded as to which health professionals should be involved in this setting. In particular, specialists generally reported that DNE involvement was still required. However, whilst some general practices have timely access to a DNE, not all do and this is the reason that an enhanced role for the PN is being proposed, in line with that occurring in other countries [19,39]. GPs working in partnership with a PN to initiate insulin for people with uncomplicated T2D, with access to specialist input if required, could help to address the issue of delays in treatment intensification and people with sub-optimal glycaemia.

GLP-1 agonists, such as exanetide, are a class of injectable hypoglycaemic agents which may be administered with oral hypoglycaemic agents. Many of the findings of this study may also be applicable to the commencement of these medications which requires patient education, including injection technique. However, the initiation of insulin represents a more complex undertaking as GLP-1 agonists are not associated with hypoglycaemia or weight gain and there is usually only a single titration step.

This work builds on previous studies which have explored the factors impacting on insulin initiation and collaborative care. Many barriers to the initiation of insulin have been identified, both in terms of patient and physician factors. Interestingly, similar themes emerge from studies investigating this all over the world and often focus on medical and training issues [40-44]. There has been less focus on the impact of health professional communication and relationships although several studies indicate that these may be important factors [45,46].

In this study role definitions for specialists were agreed but were more contentious for those in primary care, particularly practice nurses. A lack of knowledge of the practice nurse training and role were potential barriers to specialist support for a role in insulin initiation. Relational coordination appeared to be generally stronger within levels of care or where there was co-location. Lastly, personal knowledge of health professionals and their work practices were found to impact on relationships.

This qualitative study has identified a number of barriers which need to be overcome to facilitate improved relational coordination across primary and specialist care, particularly in the domains of communication and respect. Role clarification may be also be important, particularly for practice nurses.

### Need for clarity and recognition of nursing roles in primary care diabetes management

The role of the PN in Australia continues to evolve but has been hampered a lack of a clear framework to stimulate tertiary level education and knowledge development [47], funding, regulatory and interprofessional barriers [48]. A recent survey of Australian PNs showed that 2% of PNs were also credentialed DNEs, with a further 1.2% working toward attaining this qualification, whilst 59% reported providing diabetes education, assessment and management tasks on a daily or weekly basis [22]. As a result, the role of the PN can vary from practice to practice and a lack of knowledge or clinical skill related to T2D should not be assumed. Despite the concerns of some of the health professionals in this study, research has indicated that insulin initiation in primary care is safe [15-17]. Demonstrating this may be an important facilitator for the development of DNE and PN collaboration. If insulin initiation in general practice becomes a routine activity, engagement with DNEs will be important to provide in depth education to patients, to act as a boundary spanner across settings to facilitate access to specialist care for those with more complex needs and to provide support and mentoring to PNs [49]. Work by McDonald carried out in an Australian rural city found similar concerns about PNs undertaking roles in routine monitoring and education which were traditionally undertaken by DNEs, particularly in relation to consistency of care [46]. This may, in part, be due to a perceived threat to professional standing and it will be important to address this concern if collaboration between these two professional groups is to be strengthened. Some Medicare Locals, government funded regional primary care organisations charged with planning and integrating health services to improve access to effective primary health care services, fund DNEs to provide outreach care to general practices, but there is currently no funding model for DNEs in other settings to provide these services.

### Need for improved quality of communication between GPs and other health professionals

This study has demonstrated that personal knowledge of health professionals and their work practices impacts on relationships. When there is a lack of personal knowledge, quality of communication is used as a marker of quality of care and may impact on trust. The quality of communication between GPs and specialists has long been an issue of concern, and this study illustrates that it continues to be so. Research also indicates that patients perceive that there is inadequate communication between health providers involved in their diabetes care. The mean reported quality of team collaboration in diabetes care by Australian patients in the DAWN study was 56.67 on a 100 point scale, and the majority of doctor and nurse respondents indicated that better communication was important in improving collaboration [45].

Common interventions aimed at improving the quality of referral and response letters have focussed on templates, standardised letters and training of health professionals [50]. Over 95% of GPs use computers in their general practice and this offers opportunities for improved referral letters through auto-population of information, however this remains dependent on the quality and completeness of the information within the medical record. Currently there is little capacity for systems to ‘talk to each other’. Australia’s new patient controlled electronic health record may offer additional opportunities for improved communication between health professionals and organisations [51].

### The “Doctor-nurse game”

Leonard Stein described the interaction between doctors and nurses in his seminal paper The Doctor-Nurse Game [52]:

“The object of the game is as follows: the nurse is to be bold, have initiative, and be responsible for making significant recommendations, while at the same time she must appear passive. This must be done in such a manner so as to make her recommendations appear to be initiated by the physician…The game requires the nimbleness of a high wire acrobat, and if either participant slips the game can be shattered; the penalties for frequent failure are apt to be severe.”

This game, and many of the analyses of the physician-nurse relationship and interactions have taken place in the hospital setting, in which the doctor has been the keeper of both institutional and symbolic power [53]. But there have been many changes. Over half of medical school entrants are now female, nurses now hold university degrees and are increasingly using new technologies. Increasing demand for medical services without a proportional increase in doctors has resulted in nurses taking the opportunity to extend their roles, including into those previously the domain of doctors [54,55]. This is the case for DNEs who hold specialised practical knowledge about insulin initiation and administration. Despite this, two of the five DNEs interviewed in this study reported ‘playing this game’ with GPs. Whilst more evidence is needed, there are positive signs that increasing focus on interprofessional education may contribute to positive outcomes for both patients and health care professionals [56]. It may also help to improve relational coordination by breaking down historical stereotypes and hierarchies.

### Strengths

This study utilised purposive sampling in order to gain a wide range of experience and perspectives from the health professional participants. Framework analysis provided a structured, rigorous approach to analysing the resulting data with a focus on the roles and relationships between health professionals involved in the initiation of insulin for people with type 2 diabetes in the general practice setting.

### Limitations

Limitations to the findings of this study include the small numbers of health professionals interviewed and that they were recruited from a convenience sample of survey respondents, who were more likely to be involved in insulin initiation, work in private settings and be involved in diabetes or primary care research. All participants in the study were also aware of the first author’s background as a GP and this may have resulted in some socially desirable responses. Finally, this study focussed on roles and relationships between health professionals and how these impacted on insulin initiation in general practice. As a result it did not specifically explore the impact of patient factors on this task which are also important to consider.

## Conclusion

Moving away from a vertical hierarchy is required to facilitate the collaborative, multidisciplinary care which is recommended for chronic disease management. This will require a change in focus away from historical and gendered roles and professional silos to one which celebrates a common commitment/shared goals and a focus on performance, positive intent and mutual respect for each other and the setting in which each works [25]. This study has shown that whilst insulin initiation is generally recognised as acceptable in general practice, the role of the DNE and PN in this space and improved communication between health professionals are factors which need to be addressed. This will require a combined effort to evolve practices so that these are more closely aligned with the population’s changing clinical requirements. Failure to do so may result in barriers to the implementation of new models of care to the detriment of patients.

Creating structures which facilitate increased relational coordination may be important for the sustainability of models of care which have a core role for DNE to support and mentor practice nurses and also to support GPs. This may include clarification of practice nurse training and roles, increased co-location to encourage interaction between health professionals, utilising the DNE as boundary spanner to facilitate coordination across different locations and levels of care and to consider different funding structures to encourage mentoring and support of health professionals in addition to the fee for service consultations which are the basis for health professional payments in Australia.

Overcoming the current barriers to relational coordination has the ability to create improved coordination, increased work satisfaction, and lastly, to benefit patients that attend general practice for care of their type 2 diabetes.

## Endnotes

^a^Boundary spanners “facilitate transactions and the flow of information between people or groups who either have no physical or cognitive access to one another, or alternatively, who have no basis on which to trust each other” [57].

## Abbreviations

CDE: Credentialled diabetes educator; DNE: Diabetes nurse educator; GP: General practitioner; PN: Practice nurse; T2D: Type 2 diabetes.

## Competing interests

JM-N has received honoraria from Sanofi to present education sessions for practice nurses. Sanofi had no input into the content of these sessions. She has also received funding to attend educational meetings from Sanofi and Merck Sharp & Dohme. JM-N is supported by a National Health and Medical Research Council (NHMRC) postgraduate scholarship. JF, IB, DY, DO and EP are investigators on a randomised controlled trial testing a model of care for increasing insulin in general practice. This trial is principally funded by the NHMRC. Sanofi have provided insulin starter packs for this study and Roche have provided blood glucose meters and funding for the study DNE. JF, IB, DY, DO have been investigators on a pilot study for insulin initiation in general practice which received financial and/or material support from Sanofi, Medtronic, Abbott and the NHMRC Centre for Research Excellence in Diabetes.

## Authors’ contributions

JM-N conceived the study, wrote the interview schedule, interviewed the participants, analysed the data and wrote the initial draft. JF reviewed two of the interviews with JM-N. Both JF and IB participated in the design of the study and helped to draft the manuscript. DY, DO and EP provided feedback regarding the study design and draft manuscript. All authors read and approved the final manuscript.

## Authors’ information

JM-N is a GP and PhD candidate at the University of Melbourne. JF is a GP and Senior Research Fellow and IB is Research Fellow at the University of Melbourne. DY is a GP and Professor of General Practice at the University of Melbourne. All three are JM-Ns PhD supervisors. DO is an endocrinologist and Associate Professor, Department of Medicine at St Vincent’s Hospital in Melbourne. EP is Professor of Nursing and Head, School of Health Sciences at the University of Melbourne.

## Pre-publication history

The pre-publication history for this paper can be accessed here:

http://www.biomedcentral.com/1471-2296/15/20/prepub
